# Hypervelocity Impact Detection and Location for Stiffened Structures Using a Probabilistic Hyperbola Method

**DOI:** 10.3390/s22083003

**Published:** 2022-04-14

**Authors:** Sunquan Yu, Chengguang Fan, Yong Zhao

**Affiliations:** College of Aerospace Science and Engineering, National University of Defense Technology, Changsha 410073, China; yusunquan12@nudt.edu.cn (S.Y.); zhaoyong@nudt.edu.cn (Y.Z.)

**Keywords:** acoustic emission, passive sensing, impact detection, stiffened aluminum plate

## Abstract

Hyper-velocity impact (HVI) caused by a collision between orbital debris and spacecraft exists widely in outer space, and it poses a threat to spacecraft. This paper proposes a probabilistic hyperbola method based on Lamb waves analysis to detect and locate the impact in stiffened aluminum (Al) plates. A hybrid model using finite element analysis (FEA) and smoothed particle hydrodynamics (SPH) was developed to gain an insight into characteristics of HVI-induced acoustic emission (AE) and shock wave propagation. In addition, an experimental validation was carried out with a two-stage light gas gun, giving an aluminum projectile a velocity of several kilometers per second. Then a quantitative agreement is obtained between numerical and experimental results, demonstrating the correctness of the hybrid model and facilitating the explanation of received AE signals in experiments. Signal analysis shows that the shock wave quickly converts to a Lamb wave as it propagates from the HVI spot, and the zeroth-order symmetric wave mode (S0) dominates wave signal energy. The S0 wave is dispersive and shows a wide frequency range, with dominant magnitudes below 500 kHz. Finally, the HVI experiment results obtained with a light gas gun showed that the average location error could be less than 1 cm with only four sensors for a 1-square-meter stiffened metal plate.

## 1. Introduction

Meteoroids and the growing amount of orbital Debris (MODs) pose a severe threat to the on-orbit safety of spacecrafts, and most of them are difficult to be effectively monitored [1,2]. Hyper-velocity impact (HVI) is a scenario involving an impact velocity above one kilometers per second, and the relative rate between space debris and spacecraft ranges from 0 to 15 km/s. An impact sensor network on a spacecraft could be designed to monitor these events. In the space debris impact on spacecraft, due to the high relative velocity and high impact kinetic energy, elastic-plastic deformation, liquefaction, and even gasification occurs locally at the impact spot. Upon impact, dynamic strain waves known as Lamb waves are generated [3,4].

Several passive sensing techniques have been developed to locate the impact of these recorded signals captured by piezoelectric sensors [5,6,7,8]. However, the sealed cabin of the spacecraft is usually equipped with a protective screen. Its wall has many protrusions such as stiffener, spacer, and weld, making the signal characteristics and propagation law more complex. The grid-stiffened plate is commonly used as the outer wall structure of spacecraft, which is conducive to both weight reduction and strength enhancement. However, stiffeners dramatically increase the difficulty of impact detection caused by collisions between space debris and spacecraft [9,10,11,12,13].

Various methods are proposed in the literature to determine the impact force and its position on the stiffened plates [14,15]. These methods can be divided into model-based methods, transfer-function-based methods, and Artificial Neural Network (ANN) methods [16,17,18,19,20,21]. The first class of procedures depends on the analysis or numerical model of the structure. In general, Impact location is determined by minimizing the difference between the modeled response and actual response [22]. Qi et al. proposed an impact location algorithm based on posterior probability correlation. Their analysis of experiment results showed that the average location error could be 2.57 cm with proper sensor network schemes [17]. Schaefer used the intersection of several circles to locate impact spots with a least-squares method for the minimization of the error, and the impact locations fall within a circle with a radius of 34 mm [23].

It should be noted that in the model-based approach, the model must be solved many times to minimize the error function. Therefore, these methods may not be suitable for the real-time impact identification of large and complex structures. Another shock identification method is based on the transfer function approach. A relationship is established between the impact force and the response, which is then used to determine the impact [24]. ANN is used in many studies on collision recognition [21]. ANN is a mathematical model, which can be trained to simulate a complex nonlinear relationship between input and output. However, ANN needs a lot of training data, which is too expensive for HVI localization in space.

To obtain a complete understanding of HVI phenomena, some scholars proposed a variety of simulation methods [3,6,25,26,27,28,29]. Menglong et al. formulated a hybrid model to simulate the waves propagating in the vicinity of the HVI spot using three-dimensional SPH and the rest of the target structure using FEA to reap the respective merits of SPH and FE [3,6]. Ryan obtained the bending wave under HVI through fluid numerical simulation and established the fitting formula of the approximate waveform and also verified their formula’s validity by conducting an experimental study of aluminum alloy and honeycomb plates in follow-up research [30].

**Figure 1:**
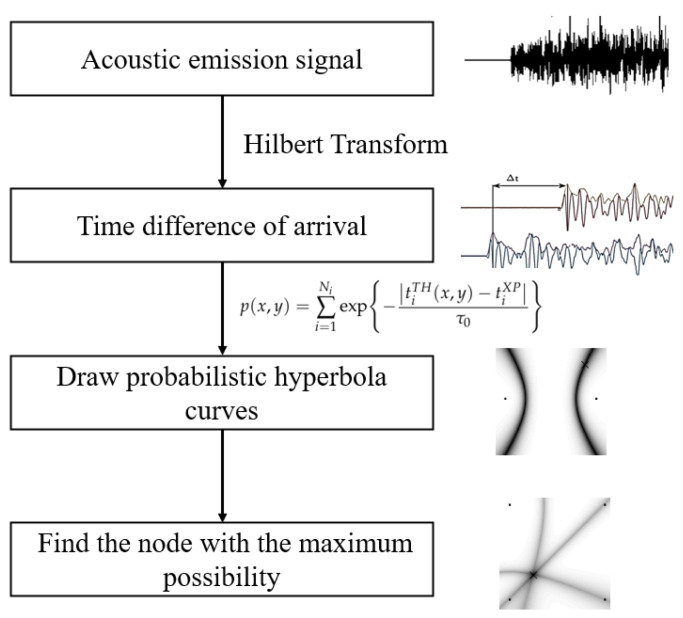
The overall diagram of the proposed impact location framework.

This paper proposes the probabilistic hyperbola localization method to locate impact events in a grid-stiffened aluminum plate. The underlying discrete probability imaging method is widely used in damage location. Often, the damage is determined by intersecting of a set of probabilistic elliptic curves [31,32]. In this paper, we propose a modified method to locate the HVI spot that uses a group of probabilistic hyperbola curves. As Figure 1 shows, first, a Hilbert transformation is used to obtain the envelope of acoustic emission signals collected by transducers. Then, the time of arrival (ToA) can be extracted when the first peak appears. Based on the location principle, which uses the time difference of arrival (TDoA), a series of hyperbolas can be gathered. In order to achieve probabilistic imaging, the observation surface is discretized to provide each point a pixel value. The probabilistic value of each node depends on the distance from those hyperbolas. Finally, the predicted impact location is determined as the node, which has the maximum possibility. This method avoids solving a set of quadratic equations, which sometimes cannot be solved when measurement errors exists.

The remainder of the paper is organized as follows: In Section 2, the localization method for HVI spot is described. Section 3 presents the cases herein investigated: a hybrid model to simulate the waves propagating in the vicinity of the HVI spot using three-dimensional SPH and the rest of the target structure using FEA to reap the respective merits of SPH and FE, together with the wave velocity estimation for stiffened plates. Finally, the experimental results are discussed in Section 4, focusing on the localization accuracy of our method. Section 5 concludes the paper.

## 2. Localization of the HVI Spot Using the Probabilistic Hyperbola Method

### 2.1. Probabilistic Hyperbola Method

**Figure 2:**
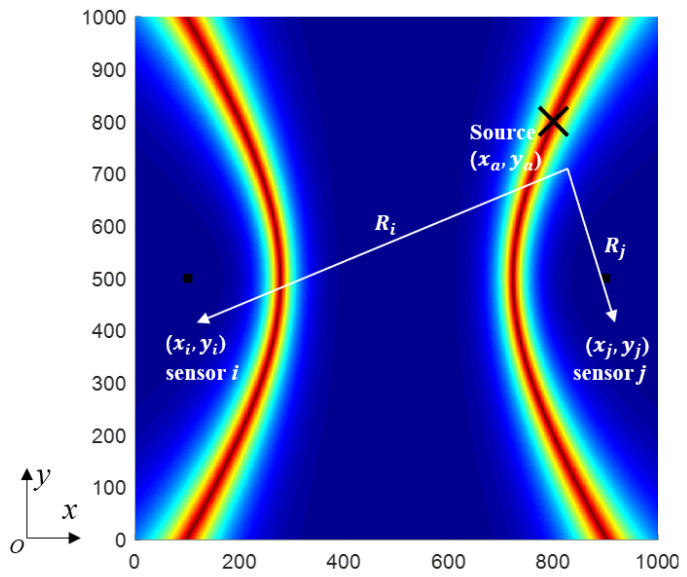
A schematic to describe the impact localization using the probabilistic hyperbola method.

Consider a pitch-catch configuration with an impact source, $\left(x_{a},y_{a}\right)$, and two receivers, $S_{i},\left(x_{i},y_{i}\right)$ and $S_{j},\left(x_{j},y_{j}\right)$, attached on a plate as shown in Figure 2. The difference between the distances $R_{i}$ and $R_{j}$ can be estimated from the distance between the traveling times of the received signals and a constant velocity of wave propagation. The locus of the possible impact locations forms a hyperbola with sensor i and sensor j as the foci and can be expressed as follows:

(1) $$\left|\sqrt{{\left(x_{a}-x_{j}\right)}^{2}+{\left(y_{a}-y_{j}\right)}^{2}}-\sqrt{{\left(x_{a}-x_{i}\right)}^{2}+{\left(y_{a}-y_{i}\right)}^{2}}\right|=TDoA_{i-j}\times V_{g},$$

where $V_{g}$ is the group velocity of the HVI induced wave, which depends on the product of the center frequency and the thickness of the plate. The time difference of arrival (TDoA) between sensor *i* and sensor *j*, $TDoA_{i-j}$, can be estimated using the Hilbert transformation, as shown in Section 2.2.

**Figure 3:**
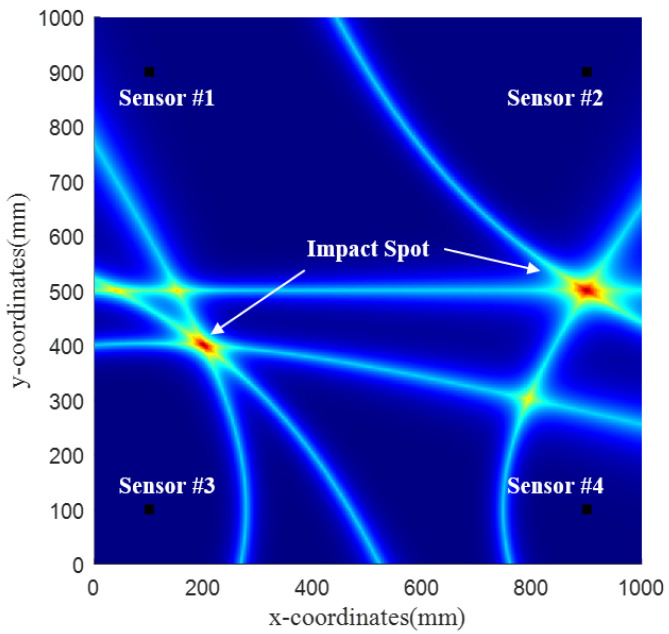
Multipoint localization based on the probabilistic hyperbola method.

Figure 3 shows a diagram of locating two acoustic emission points by using the probabilistic hyperbola method. It can be seen that, when there are multiple impact points at the same time or when numerous impact events occur in less than one sampling period, the probabilistic hyperbola method can still clearly show their location.

In order to achieve probabilistic localization imaging, the observation surface is discretized to provide each point pixel value. Assuming each node in the plate as a possible impact spot, the theoretical TDoA can be computed at each node (x,y). This procedure is repeated for each node and each impact-sensor path, and an impact index, p(x,y), at any node (x,y) is obtained by comparing the theoretical TDoA, $t_{i}^{TH}\left(x,y\right)$, to the data extracted from the scattered signal, $t_{i}^{XP}$, as follows:

(2) $$p\left(x,y\right)=\sum_{i=1}^{N_{i}}\exp\left\{-\frac{\left|t_{i}^{TH}\left(x,y\right)-t_{i}^{XP}\right|}{\tau_{0}}\right\},$$

where $N_{i}$ is the total number of sensors, and $\tau_{0}$ is a decay factor as the decay rate of an exponential windowed function; its value is fixed at 0.01 ms in this study by adjusting the display effect many times. The estimated impact location is then chosen as the point having the maximum value of p(x,y).

### 2.2. TDoA Estimation Using Hilbert Transformation

To estimate TDoA, which corresponds to the time taken by the wave packet to travel from an impact spot to the sensors through the different paths, the Hilbert transformation algorithm was introduced. The technique assumes that the input signal, *x*, is a finite block of data. This assumption allows the function to remove the spectral redundancy. Time of arrival estimation by the Hilbert Transformation uses a five-step algorithm.

Step 1: Calculate the FFT of the input sequence, x[n],0≤n≤N−1, storing the result in a vector X(f):
  (3) $$X\left(f\right)=T\sum_{n=0}^{N-1}x\left[n\right]\exp\left(-j2\pi fnT\right),$$
  where *f* is the frequency, and *T* is the sample period. Create a vector *h* for which its elements h(i) have the following values.
  (4) $$h\left(i\right)=\left\{\begin{array}{c} 1,i=1,(N/2)+1\\ 2,i=2,3,\ldots ,(N/2)\\ 0,i=(N/2)+2,\ldots ,N \end{array}\right.,$$

Step 2: Calculate the element-wise product of *X* and *h*.
  (5) Z(i)=X(i)×h(i),0≤i≤N−1,

Step 3: Calculate the inverse FFT of the sequence obtained in step 2 and return the first *N* elements of the result.
  (6) $$z\left(n\right)=\frac{1}{NT}\sum_{m=0}^{N-1}Z\left[m\right]\exp\left(+j2\pi mn/T\right),$$

Step 4: Calculate the absolute value of the sequence obtained in step 3.
  (7) $$y\left(n\right)=\left|z\left(n\right)\right|,$$

Step 5: Once the envelope of the waveforms has been obtained by Hilbert transformation, the time of arrival can be extracted when the first peak appears.
  (8) $$ToA=\min\{t_{peak-0},t_{peak-1},\;\ldots ,t_{peak-n}\},$$

## 3. Numerical Investigation and Demonstration

### 3.1. Modeling Shock Waves under HVI

The phenomenon of HVI is complex, which involves the material properties, strength behavior, viscosity, and thermal effect. This paper uses a hybrid model with FEM and SPH to simulate HVI and shock wave propagation. The instantaneous local pressure produced by HVI is exceptionally high, far greater than the strength of the material. The material will appear similarly to a fluid, and the typical debris cloud diffusion phenomenon will occur after HVI [6]. The SPH method has a strong advantage in describing these two phenomena, so the SPH method has become the primary method used in HVI research. However, the SPH method has tensile stability, so some scholars have proposed SPH and Lagrange grid hybrid algorithm [3]. The SPH method is used in the collision center region where the impact pressure is very high, and the material is similar to a fluid. In contrast, the Lagrange grid method is used far away from the center, where material strength plays a decisive role. In this way, the calculation efficiency and accuracy can be significantly improved. Equation of state (EOS), metal plasticity model, and failure criteria are necessary to describe the behavior of materials under HVI [26].

Two classical EOS models are Mie-Gruneisen and Tillotson model, and the Tillotson model is usually used to deal with the problems with phase transformation. However, phase transformation rarely occurs for aluminum alloy. Thus, the Mie–Gruneisen model is selected for solid shock wave analysis. A Mie–Grüneisen EOS is linear in energy. The most common form is as follows:

(9) $$\left\{\begin{array}{c} p-p_{H}=\Gamma \left(e-e_{H}\right)\\ p_{H}=\frac{\rho_{0}c_{0}^{2}\mu \left(1+\mu \right)}{{\left[1-\left(s-1\right)\mu \right]}^{2}}\\ e^{H}=\frac{p_{H}}{2\rho_{0}}\left(\frac{\mu }{1+\mu }\right),\mu =\frac{\rho }{\rho_{0}}-1\\ \Gamma \rho =\Gamma_{0}\rho_{0}=\;\mathrm{const}\;,U=c_{0}+su_{p} \end{array}\right.,$$

where $p_{H}$ and $e_{H}$ are the Hugoniot pressure and specific energy (per unit mass) and are functions of density only, and Γ is the Grüneisen ratio defined as $\Gamma =\Gamma_{0}\rho_{0}\rho$, where $\Gamma_{0}$ is a material constant and $\rho_{0}$ is the reference density, and *p* and *e* are correlated with density and internal energy. $c_{0}$ is the bulk speed of sound, and *s* is the slope of the linear Us-Up Hugoniot form of the EOS. Material parameters $c_{0}$ and *s* define the linear relationship between the shock velocity, Us, and particle velocity Up.

Metal plasticity models are intended for crash analyses, metal forming, and general collapse studies. One of the classical metal plasticity models, the Johnson–Cook plasticity model, is chosen in this paper, which is particularly suited for modeling high-strain-rate deformation of metals. This model is a particular type of Mises plasticity that includes analytical forms of the hardening law and rate dependence. Johnson–Cook hardening is a specific type of isotropic hardening where the static yield stress, $\sigma_{0}$, is assumed to be of the following form:

(10) $$\sigma_{0}=\left(A+B{\left(\varepsilon_{p}\right)}^{n}\right)\left(1-{\hat{T}}^{m}\right),$$

where $\varepsilon_{p}$ is the equivalent plastic strain and *A*, *B*, *n*, and *m* are material parameters measured at or below the transition temperature, $T_{\mathrm{room}}$. The nondimensional temperature is defined as follows:

(11) $$\hat{T}≜\left\{\begin{array}{ccc} 0 & \;\mathrm{for}\; & T<T_{\mathrm{room}\;}\\ \left(T-T_{\mathrm{room}\;}\right)/\left(T_{\mathrm{melt}\;}-T_{\mathrm{room}\;}\right) & \;\mathrm{for}\; & T_{\mathrm{room}\;}\leq T\leq T_{\mathrm{melt}\;}\\ 1 & \;\mathrm{for}\; & T>T_{\mathrm{melt}\;} \end{array}\right.,$$

where $T_{\mathrm{melt}}$ is the melting temperature. When $T>T_{\mathrm{melt}}$, the material will be melted and will behave like a fluid, so there will be no shear resistance since $\sigma_{0}=0$. Table 1 provides the values of A,B,n,m, $T_{\mathrm{room}}$, and $T_{\mathrm{melt}}$ as part of the metal plasticity material definition.

The failure model is used to define the ultimate tensile stress, and for 2017 and 5A06 aluminum alloy, they are 2.5 GPa and 1.8 GPa, respectively. However, for SPH numerical simulation, since the computational domain of the problem is realized by filling SPH particles, it is unnecessary to consider the material failure behavior and failure model.

### 3.2. Numerical Implementation

To illustrate the effectiveness of the proposed damage localization method, a numerical study for a stiffened aluminum plate is performed. The material properties of the aluminum plate are shown in Table 1 and Table 2. To simulate HVI phenomenon, the commercial finite element package ABAQUS is used. An explicit central difference scheme is employed to obtain the responses of the plate [33].

**Figure 4:**
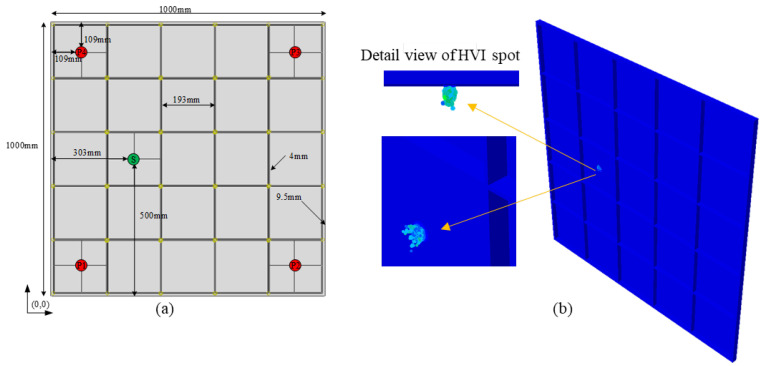
(**a**) Schematic diagram of the transducers configuration in simulation and experiment and (**b**) a screenshot of simulation, $t=4.5\times 10^{-5}$ s.

As shown in Figure 4a, the size of the Al plate is 1000 mm × 1000 mm × 2.5 mm. The height and width of quadrilateral grid stiffeners are 20 mm and 4 mm, respectively, and the spacing between two adjacent stiffeners is 200 mm. The origin of the coordinate system is set at the lower-left corner of the plate. Assume that one impact event and four sensors are configured on the plate. All the boundaries are set as displacements fixed in the model.

Simulation for the normal HVI scenario was performed for 0.001 seconds since the impact initial. A snapshot recording the HVI produced debris clouds at typical instants within this duration is exhibited in Figure 4b.

**Figure 5:**
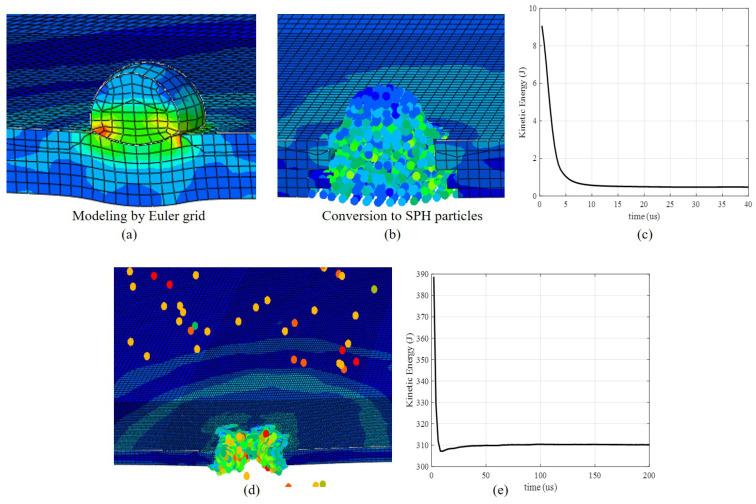
Local screenshot of simulation with a 3.8 mm projectile. (**a**) The Euler grid can still work well under low-velocity impact with a 500 m/s projectile; (**b**) conversion Euler grid to SPH particles when the strain rate is above 0.01; (**c**) the curve of Kinetic energy with time with the velocity of a projectile is 500 m/s; (**d**) the target plate is penetrated with the speed of a projectile is four km/s; (**e**) the curve of Kinetic energy with time with the velocity of a projectile is four km/s.

For the single-layer plate impact model established by the SPH method, when the total energy of the target plate has stabilized, it can be determined whether the single-layer plate is penetrated by observing whether there is obvious material peeling off on the back of the target plate or whether the projectile particles penetrate the target plate. Figure 5 shows a local screenshot of the target plate. The size of the projectile is 3.8 mm, and the thickness of the target plate is 2.5 mm. The projectile velocity in Figure 5a is 500 m/s, and the plate is not penetrated. As a result, a pit is formed on the upper surface of the plate and a bulge is formed on the lower surface. The impact velocity of the projectile in Figure 5b is four km/s. Under such conditions, the plate is penetrated, and a hole is formed. The simulation results show that the critical velocity of perforation is 1.1 km/s under the above parameters, which is consistent with our later experimental results in Section 4.

### 3.3. Wave Velocity Estimation for Stiffened Structure

**Figure 6:**
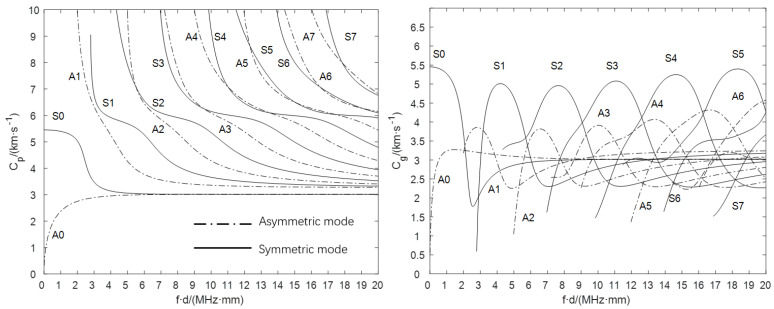
(**left**) Phase velocity ($C_{p}$) and (**right**) group velocity ($C_{g}$) of the 5A06 brand aluminum alloy plate under different frequency (*f*) and plate thickness (*d*).

The acoustic waves generated by impact usually exist in the form of P-waves and S-waves in the solid material. When the waves propagate in the thin plate material, they will reflect and interact with each other continuously at the upper and lower boundaries, forming a new wave package and propagating forward in the plate. The new wave package becomes the guided wave, also known as the Lamb wave. Similarly to most guided waves, Lamb waves are dispersive, and their velocity depends on the frequency of the wave and the thickness of the plate. This phenomenon is called dispersion. Dispersion curves describe and predict the relationship among frequency, phase velocity, group velocity, and thickness [32,34]. As an example, the dispersion curve of the aluminum plate is shown in Figure 6.

**Figure 7:**
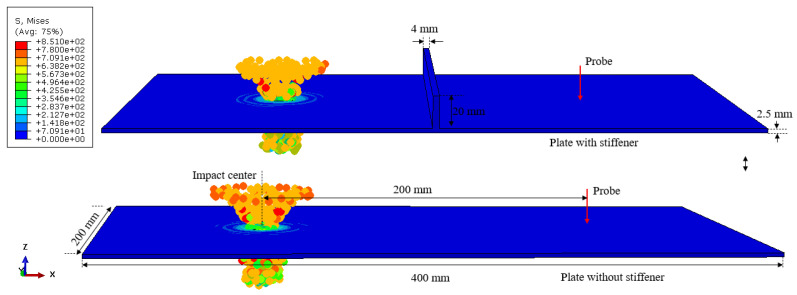
A screenshot of the numerical simulation model; velocity = 4 km/s; $t=1.6\times 10^{-5}$ s.

Figure 7 shows the HVI numerical simulation models with stiffener and also a plate without stiffener as comparison. The size of the Aluminum plate is 400 mm × 200 mm × 2.5 mm. The height and width of stiffener are 20 mm and 4 mm, respectively. The distance between impact center and probe is 200 mm.

**Figure 8:**
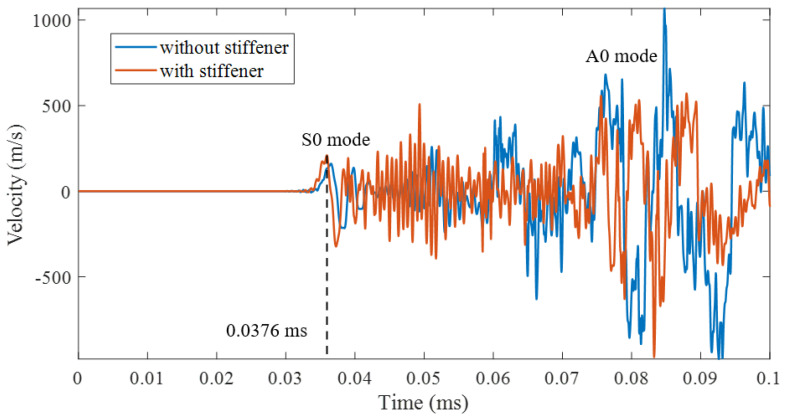
Simulated velocity comparison between flat plate and stiffened plate.

As Figure 8 shows, the TDoA value for the impact spot and probe is $3.76\times 10^{-5}$ s, and the distance between them is 200 mm as Figure 7 shows. Then, we can obtain the S0 mode Lamb wave propagation velocity in the numerical simulation as 5324.7 m/s.

**Figure 9:**
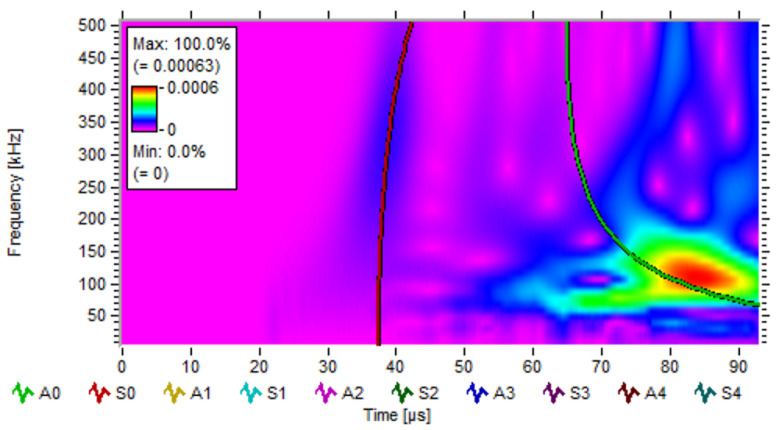
Wavelet transforming diagram of the signal in stiffened plate.

Figure 9 shows the continuous wavelet transform (CWT) of the signal in stiffened plate. The CWT is a time-frequency transform, which is ideal for analyzing nonstationary signals. A signal being nonstationary means that its frequency-domain representation changes over time. From Figure 9, its obvious that only S0 and A0 modes show in our simulation. The thickness of plate is 2.5 mm in our simulation, and the signal frequency of S0 mode is about 350 kHz, as shown in Figure 9. Then, we can obtain the product of the frequency, and the thickness is about 0.875 MHz·mm, and the phased velocity and group velocity are about 5300 km/s, which is consistent with the theory’s results, as Figure 6 shows.

As Figure 8 shows, the stiffeners significantly attenuate the shock wave. Furthermore, the stiffener reflects the signal many times, making the signal look more chaotic. The stiffener can transform the shock wave signal containing multiple modes into S0 mode of Lamb wave, which can be described by S0-related attributes.

## 4. Experimental Validation

### Experimental Setup

**Figure 10:**
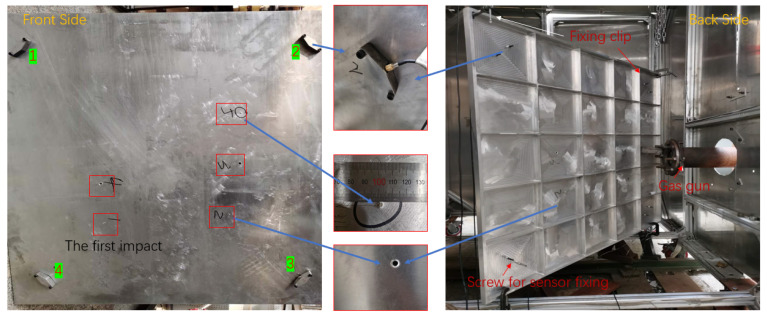
Experimental setup for the impact test of a plate structure.

To further investigate the effectiveness of the proposed method in practical applications, experimental studies using a two-stage light gas gun (LGG) are conducted. LGGs are often used to simulate HVI events of space debris [23,26]. As Figure 10 shows, the aluminum alloy-stiffened plate in the typical spacecraft structure was taken as the research object, and the size was 1000 mm × 1000 mm × 2.5 mm. Two clamps and two weight blocks were used to hang the test piece in order to prevent it from shaking violently. Four sensors were installed on the smooth side of the stiffened plate and arranged anticlockwise with the plate surface as the origin. The installation positions of the four transducers sensors are consistent with Figure 4. Special clamps are manufactured to fix the sensors. The 2017 aluminum alloy ball projectile with a diameter of 3.18 mm was used, and 30,000 data points were collected by data recorder with sampling rate of 6 million samples per second.

**Figure 11:**
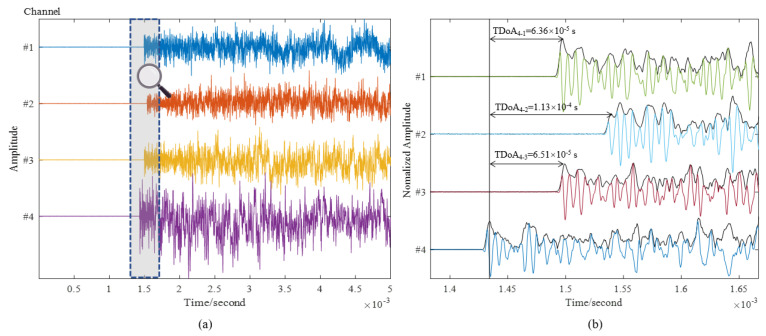
Signal waveform captured by experiment. (**a**) All signal wave with the length of 30,000 points; (**b**) Data intercepted when the signal arrives nearby with the length of 1700 points.

Figure 11a is the original signal waveform generated in the first impact event. The signal amplitude of channel 4 is larger than that of the other channels, indicating that the impact position is closer to channel 4, which is consistent with the actual impact position (298, 307). Figure 11b is the normalized waveform signal with the amplitude envelops by Hilbert transforming. The delay times from sensor 4 to 1, 2, and 3 are $6.36\times 10^{-5}$, $1.14\times 10^{-4}$, and $6.51\times 10^{-5}$ seconds, respectively. The normalized signal is obtained by dividing the original signal by the maximum value, which is easier to define a threshold for detecting signal arrival time. In addition, the arrival time of the signal is only related to the shape of the wave, so normalization will not change the arrival time of the signal.

When the signal arrival time is determined by using the envelope of the impact acoustic emission signal, the moment where the first peak value is taken as the signal arrival time can reduce the error caused by the traditional threshold method to determine the wave’s velocity. By using the first peak time, positioning accuracy can be improved. It is difficult to accurately determine the arrival time of the S0 mode wave due to the reflection effect in our traditional impression. However, the arrival time of the S0 wave can be accurately determined by Hilbert transformation technology.

**Figure 12:**
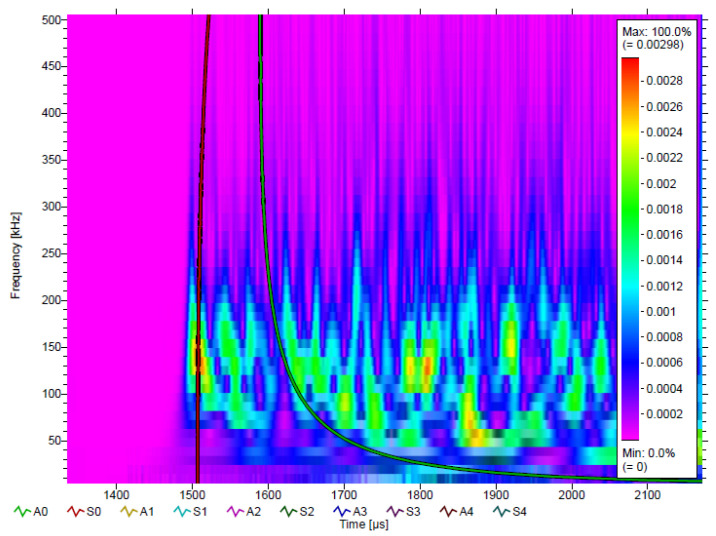
Wavelet transforming diagram of the experimental signal, velocity = 3.97 km/s.

Figure 12 shows that the signal spectrum is below 500 kHz, and only A0 and S0 modes are excited, which is consistent with the frequency of the lead breaking signal. The lead breaking experiment is also used to evaluate the positioning effect of the acoustic emission system in our investigation. Experimental results show that the acoustic signal characteristics of spacecraft impacted by debris are consistent with those of lead-breaking signals.

**Figure 13:**
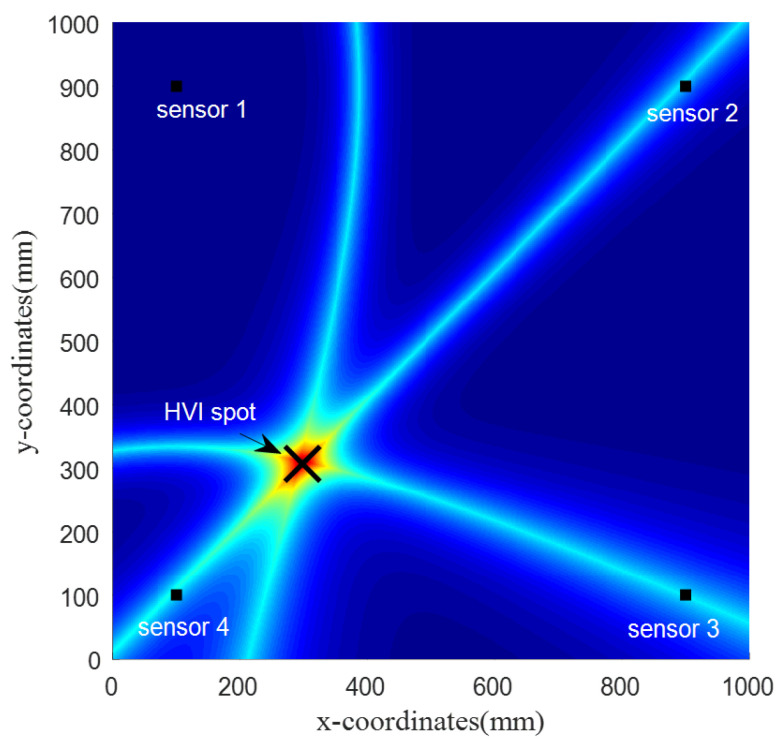
The probabilistic results of the impact location estimate for validation.

Figure 13 shows the localization result using the probabilistic hyperbola method. The predicted impact spot is at (298, 307) mm, 3 mm away from the actual impact position.

**Figure 14:**
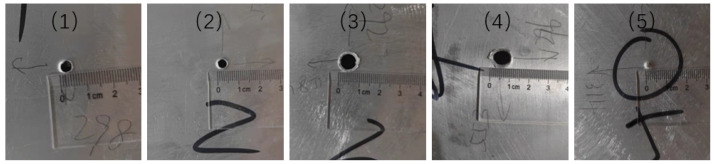
The photos of hypervelocity impact results.

A total of five experiments were carried out, as shown in Table 3 and Figure 14. The maximum error of the experiment is 12.08 mm, and the average error is 6.02 mm, which is enough for practical applications. The first four groups of experiments caused perforation, and the last group failed to penetrate the aluminum alloy plate and created an impact crater. The results show that when the diameter of the projectile and the thickness of the target plate are fixed, the projectile’s velocity is the main factor affecting the damage mode and the degree of the target plate.

The aluminum plate was tilted 45 degrees in the fourth group to observe the difference between oblique impact and vertical impact. In the process of actual spacecraft in orbit, most of them are inclined to impact, while the normal impact is only a particular case. The hole caused by the oblique impact is larger than that caused by normal impacts, but the influence on positioning results may be ignored.

## 5. Conclusions

This paper has focused on the impact detection and location in the stiffened aluminum plate using Lamb waves. Based on the research and development of space debris on orbit sensing systems, the acoustic emission phenomena of hypervelocity impact and on-orbit sensing technology are systematically studied in this paper. A model has been built to reveal the mechanics of Lamb waves crossing the stiffener. The acoustic emission (AE) signal generated by hypervelocity impact is obtained using a two-stage light gas gun experiment. By using the probabilistic hyperbola method proposed in this paper, the maximum error of the impact location is 12.08 mm, and the average error is 6.02 mm for a 1-square-meter plate, which is sufficient for practical applications.

The influence of stiffeners on the acoustic emission signal characteristics is obtained by numerical simulation. The arrival time determination method of acoustic emission signal based on waveform and mode is given, respectively, to realize the location of the hypervelocity impact source of an aluminum alloy-stiffened plate. When the wave meets the stiffener, penetration and reflection will occur. The simulation results show that the velocity of the S0 mode wave is less affected by the stiffener, and its propagation in the stiffened plate can be regarded as isotropic. The location of the impact source can be realized by using the arrival time of the S0 mode wave and the location method of a flat plate, which are verified by the FEA and hypervelocity impact tests, respectively.

Future work includes larger, more complex structures, wireless sensor development, and the assessment of impact-induced damage, since the sensors cannot resist a hypervelocity impact and are redundant in terms of reliability. The number and layout of sensors are also interesting directions for exploration.

## Figures and Tables

**Table 1:** Material properties of Johnson–Cook strength model.

Brand	*A* (GPa)	*B* (GPa)	*n*	*C*	*m*	$T_{\mathrm{melt}}$	$T_{\mathrm{room}}$
2017	0.26	0.426	0.34	0.0015	1.0	786	300
5A06	0.27	0.426	0.34	0.0015	1.0	844	300

**Table 2:** Material properties of state equation.

Brand	Density (${g/\mathrm{cm}}^{3}$)	$c_{0}$ (m/s)	s	$\Gamma_{0}$
2017	2.79	5328	1.338	2
5A06	2.64	5328	1.338	2

**Table 3:** Error analysis for the different locations (unit: mm).

No.	Velocity	Direction	Impact Location	Calculated Location	Error	Results	Radius
1	1.26	0°	298, 307	301, 310	4.24	hole	5
2	1.31	0°	665, 325	670, 336	12.08	hole	5
3	3.91	0°	708, 516	703, 517	5.10	hole	9
4	3.97	45°	296, 447	292, 451	5.66	hole	11
5	0.81	0°	709, 686	706, 686	3.00	crater	3

## Data Availability

Not applicable.
